# Increased occurrence of ACL injuries for football players in teams changing coach and for players going to a higher division

**DOI:** 10.1007/s00167-021-06604-w

**Published:** 2021-05-13

**Authors:** Alexander Sandon, Werner Krutsch, Volker Alt, Magnus Forssblad

**Affiliations:** 1 Department of Molecular Medicine and Surgery, Stockholm Sports Trauma Research Center, Karolinska Institute, Stockholm, Sweden; 2 Department of Trauma Surgery, University Medical Center Regensburg, Regensburg, Germany

**Keywords:** ACL, ACL injury, Football, Soccer, Return to sports

## Abstract

**Purpose:**

To identify football-specific factors associated with ACL injuries that can be targeted for sport-specific injury prevention.

**Methods:**

A study-specific questionnaire was developed to study the characteristics of ACL injuries in football including intrinsic, extrinsic, and injury specific factors. The questionnaire was available at the Swedish national knee ligament registry’s website for the football players to voluntarily fill out. Data are presented on group level for all football players in total and for females and males separate to examine gender-specific differences. The results are based on answers collected over a 3-year period from 2875 football players, 1762 (61%) males and 1113 (39%) females.

**Results:**

ACL were more frequently sustained during games 66% than during practices 25%. The injury mechanism was non-contact in 59% and contact in 41%. For the contact injuries during games, no action was taken by the referee in 63% of the situation and a red card was shown in 0.5%. The risk of ACL injury was highest early in the football game with 47% sustained during the first 30 min and 24% in the first 15 min. Players changing to a higher level of play 15% had a higher rate of ACL injuries than players changing to a lower level 8%. This difference was especially seen in female football players with 20% of ACL injuries being sustained by players going to a higher division compared to 7% for those going to a lower division. 15% of the male and 21% of the female ACL injuries occurred in teams with a coach change during the season. Knee control exercises to warm up was used by 31% of the female players and 16% of the males. 40% of the players reported that they did not plan on returning to football.

**Conclusion:**

Neuromuscular training programs have proven to reduce ACL injuries, but greater adherence to these remains a challenge as only 1 in 5 of the ACL-injured football players report using them. Teams changing coach and players going to a higher division appear to have an increased risk of ACL injury warranting attention and further investigations.

**Level of evidence:**

IV.

## Introduction

Football is a physically demanding sport involving high-speed runs, pivoting activities, and contact situation that might result in an injury. A recent long-time follow-up of professional football identified a wide range of football related injuries. One of the most severe injuries with the longest lay off from football was an Anterior Cruciate Ligament (ACL) injury [8]. ACL injuries are often viewed in the light of how it affects the players’ football career. Reconstructive surgery is often performed to restore knee stability and to allow a return to football. For professional players, the lay of from football is most cases more than 6 months, but the return rate is very high [16, 27]. In amateurs, the return rate is considerably lower and only just over half the players return to football [4, 21]. Another aspect is how football affects the future knee health of the player. A recent study following the most talented youth players in Sweden found that as many as 10% of the players underwent ACL reconstruction during the follow-up period [22]. In the Scandinavian knee ligament registries, the cause of the ACL injuries is football in about 40% of patients [11]. The risk of developing osteoarthritis is high after an ACL injury [2], and it is there for important to try to prevent and reduce the number of ACL injuries.

For the present study, a questionnaire was developed for male and female players to study the characteristics of ACL injury in football. The hypothesis was that there are football-specific factors associated with ACL injuries and the main objective of the study is to try to identify such factors that can be targeted for sport-specific injury prevention.

## Materials and methods

The research was approved by the regional ethics committee in Stockholm ID 2011/337–31/3 and Linköping ID 2020-03747.

A study-specific questionnaire was created to try to identify football-specific factors associated with ACL injuries. The questionnaire was initially developed in Germany for the national ‘German ACL Registry in Football’ and later adopted and translated into Swedish [14]. The Swedish version of the questionnaire was made available for ACL-injured patients on the website of the Swedish National Knee Ligament Registry (SNKLR) in 2017. The SNKLR was established in 2005 as a surgical registry for ACL injuries and has recently started to also include non-surgically treated patients [1]. The patients in the registry are asked to fill out patient reported outcome measures (PROMs) like the Knee Injury and Osteoarthritis Outcome Score (KOOS) and the EuroQol 5-D (EQ-5-D) before surgery and at 1, 2, 5, and 10 year follow-up [19, 20]. As the patients log on to the SNKLR to fill out these other PROMs, the present study-specific questionnaire was available for football players to voluntarily fill out. Answers to the questionnaire have been collected for 3 years.

### Study-specific questionnaire

An ambition to identify football-specific characteristics of ACL injuries resulted in the inception of the present questionnaire. The development of the questionnaire began in 2015 when an increase in ACL injuries was found after the implementation of new professional football league in Germany [17]. The suspicion was that ACL injuries in football may be influenced by many different intrinsic and extrinsic factors and that some of them might be available for intervention to reduce the number or injuries. A wide variety of different player-specific characteristics, field-specific characteristics, as well as short-term changes on players` ability and performance were analyzed in detail. All the variables in the questionnaire are presented in the Results section.

### Statistical analyses

All statistical analyses were conducted in IBM SPSS version 27. All data were anonymized before the statistical analysis. Descriptive statistics were calculated for all study variables. The data are presented on group level for all football players in total and for females and males separate to examine gender-specific differences.

## Results

The results are based on answers from 2875 football players, 1762 (61.3%) males and 1113 (38.7%) females. The mean age of the male players was 4.5 years older than for the female payers. Anthropomorphic data are available in Table 1. For both male and female players, the Body Mass Index (BMI) had a normal distribution.

**Table 1:** Anthropomorphic data of male and female anterior cruciate ligament injured male and female football players (*n* = 2875)

Variable	Male		Female	
	*n* 1762	61.3%	*n* 1113	38.7%
	Mean	SD	Mean	SD
Age (years)	28.4	7.9	23.9	9
Height (cm)	181	6.5	167	5.9
Weight (kg)	81	11.3	66	9
Body mass index (BMI)	24.9	3	23.4	2.8

For both male and female players, defenders (37.1%) and midfielders (36.8%) were the positions who sustained most of the ACL injuries and only 5.2% of the ACL-injured players were goalkeepers. The players were predominantly right footed (81.1%). Twice as many of the male football players (13.3%) were left-footed compared to the female players (6.6%). ACL injuries occur to football players at all levels. However, most of the ACL injuries were sustained by footballers playing at the lower levels (Table 2).

**Table 2:** Football-specific data for ACL-injured football players (*n* = 2875)

Variable	Total	Male	Female
	%	%	%
Position			
Goalkeeper	5.2	4.8	5.8
Defender	37.1	36.5	38
Midfielder	36.8	37.5	35.6
Forward	21	21.2	20.6
Dominant leg			
Right	81.1	79.2	84.1
Left	10.7	13.3	6.6
Two-footed	8.2	7.5	9.3
Level of play			
Swedish top division	2.8	2.1	4
Division 2	1.6	0.9	2.6
Division 3	6.6	2.1	13.7
Division 4	11.5	5.1	21.7
Division 5	14.4	8.5	23.7
Division 6	14.9	16	13.1
Division 7	13.4	19.5	3.6
Division 8 or lower	11.4	17.7	1.3
Non-league	8	10.9	3.5
Recreational	15.5	17.2	12.8

	Mean (SD)	Mean (SD)	Mean (SD)
Years played	15.8 (7.2)	17.2 (7.6)	13.6 (5.9)

ACL injuries were more common in the right leg (53.5%) than in the left leg (46.5%). For both male and female players, the ACL injury most often occurred in games (65.7%) rather than practices (24.8%) with the remaining (9.5%) happening in other types of situation for example playing recreational among friends. The males sustained more ACL injuries during home games and the female more during away games. The player reported injury mechanism was non-contact for 59.1% and contact for 40.9%. For the contact injuries during games, no action was taken by the referee in 63.1% of the situation and a straight red was shown in only 0.5% of the situation. Although most stopped playing after sustaining the ACL injury 3.9% managed to continue playing. More ACL injuries were sustained while playing on artificial grass than on natural grass. The risk of ACL injury was highest early in the football game with 47.2% sustaining the injury in the first 30 min and 23.9% already in the first 15 min (Table 3).

**Table 3:** Injury occurrence data for ACL-injured football players (*n* = 2875)

Variable	Total	Male	Female
	%	%	%
Injured leg			
Right	53.5	54.3	52.2
Left	46.5	45.7	47.8
Activity at time of injury			
Home game	33.9	34.2	33.5
Away game	31.8	27.9	38
Practice	24.8	27.1	21.2
Other	9.5	10.8	7.3
Injury mechanism			
Contact	40.9	39.9	42.5
Non-contact	59.1	60.1	57.5
Referees decision			
Red card	0.5	0.4	0.6
Yellow card	3.4	4	2.5
Freekick	12.2	12.5	11.8
Other	20.8	20.3	21.5
No action	63.1	62.8	63.6
Started the game			
Yes	86.1	85.7	86.6
Substituted on	13.9	14.3	13.3
Action after injury			
Continued playing	3.9	4.3	3.1
Stopped playing	86.6	85.3	88.8
Unsure	9.5	10.4	8.1
Pitch surface			
Grass	37.7	37.3	38.4
Artificial grass	45.7	44.8	47.2
Gravel	1.4	1.6	1
Indoor	12.1	12.8	11.1
Other	3.1	3.5	2.4
Game minute			
Mean (SD)	40.3 (26.4)	39.7 (26.1)	41.3 (26.9)
0–15	23.9	24	23.9
16–30	23.3	23.9	22.1
31–45	13.3	14.3	11.9
46–60	13.9	13.7	14.1
61–75	14.8	13.8	16.4
76–90 +	10.8	10.3	11.6

**Fig. 1:**
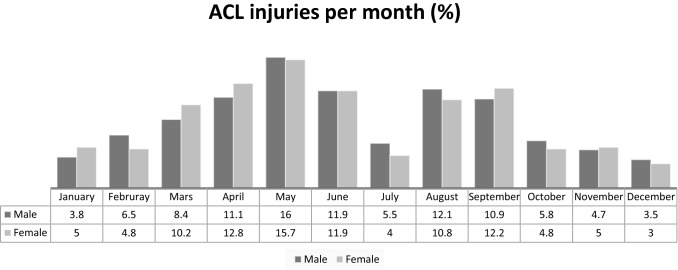
Frequencies of ACL injuries in football per month in Sweden (*n* 2619)

**Fig. 2:**
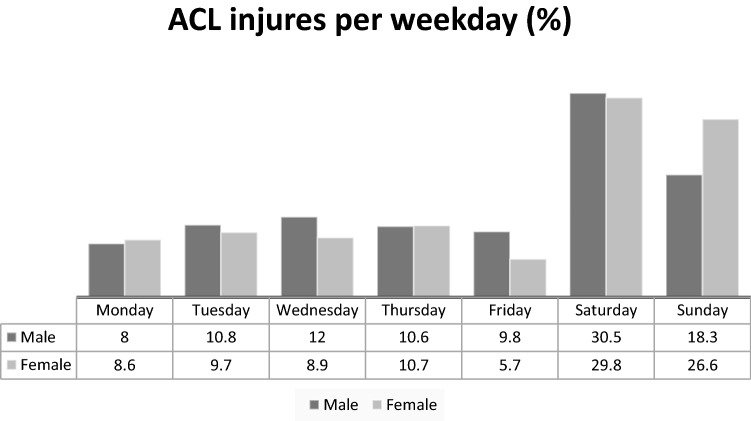
Frequencies of ACL injuries in football per weekday in Sweden (*n* 2312)

The Swedish football season is usually played from April until the beginning of November with a brake in July and the ACL injuries are more frequent in season compared to the off season with a slight peak in May (Fig. 1). Most of the ACL injuries were sustained during the weekend (Fig. 2).

Players changing to a higher level of play had a higher rate of ACL injuries than players changing to a lower level. This difference was especially seen in female football players with 19.7% of ACL injuries being sustained by players going to a higher division compared to 7% for those going to a lower division. One out of 4 of the males and 1 out of 3 of the females had a coach change before or during the season of the injury and the frequencies of ACL injuries were higher if the team changed coach during the season. In the 3 months prior to the ACL injury roughly twice as many players claim to have lost weight compared having gained weight. Knee control exercises to warm up was used by 31.4% of the female players and 15.6% of the male players. One in five reported to have had an injury or symptoms in the 6 weeks leading up to the ACL injury. A previous ACL injury was reported by 17.9% of the females and by 14% of the males. Further data and other influencing factors are found in Table 4.

**Table 4:** Preparation and influencing factors for ACL injuries in football players (*n* = 2875)

Variable	Total	Male	Female
	%	%	%
Club change for this season			
Yes	11.8	11.5	12.2
No	88.2	88.5	87.8
Division change this season			
No	77	79.3	73.3
Yes, to a higher	14.6	11.5	19.7
Yes, to a lower	8.4	9.2	7
Coach change			
No	72.3	75.5	67.3
During the season	17.3	15.2	20.8
Before the season	10.4	9.4	11.9
Weight change previous 3 months			
About the same	78.9	77	81.9
Gained weight	7.2	7.6	6.6
Lost weight	13.9	15.4	11.4
New football shoes last month			
Yes	11.8	13	10
No	88.2	87	90
Shoe type at time of injury			
Firm ground—permanent studs	62.4	63.5	60.6
Soft ground—screw-in studs	1.8	2.6	0.5
Artificial grass	19.7	16.8	24.4
Indoor	6.3	7.2	4.9
Other	9.8	10	9.5
Weather conditions			
Sun	42.5	41.5	44
Cloudy	29.3	29.7	28.5
Rain	6.8	6.9	6.7
Snow	3.7	3.9	3.4
Other	17.7	17.9	17.3
Games played in the previous 7 days			
0	37.3	35.2	40.9
1	50.4	49.4	52
2 or more	12.3	15.3	7.1
Warm up using knee control exercises			
Yes	21.7	15.6	31.4
No	78.3	84.4	68.6
Previous injury			
ACL	15.5	14	17.9
PCL	1.2	1.2	1.2
Symptoms or injury in the last 6 weeks before the ACL injury			
Yes	19.8	18.7	21.6
No	80.2	81.3	78.4
Distribution of symptoms and injuries per body part			
Head	4.2	2.7	6.2
Neck	2.5	1.5	3.8
Back	18.9	19.4	18.3
Groin	14	16.1	11.3
Hip	9.3	7.9	11.3
Thigh	16	13.6	19.2
Knee	60	62.7	56.3
Ankle/foot	25.4	23.6	27.9

The median in days until ACL diagnosis was 21, but the mean was 93 days, and this large difference was due to a few extreme outliers where the diagnosis was not made until several years after the suspected initial injury. Magnetic resonance imaging (MRI) was performed on 73.3% of the players and 54.6% had plain radiography. Associated injuries were reported by 46.7% of the players. In total, 60% of the players plan on returning to football after the ACL injury and a subanalysis found that younger players (*p* < 0.001) and players in the higher divisions (*p* < 0.001) are more likely to plan on returning to football. Surgical treatment was planned for 94.9% of the players (Table 5).

**Table 5:** Time until ACL diagnosis, imaging, associated injuries, intention to return, and treatment data for ACL-injured football players (*n* = 2875)

Variable	Total	Male	Female
Days until ACL diagnosis			
Median	21	24	21
Mean (SD)	93 (260)	106 (285)	73 (214)

	%	%	%
Imaging			
MRI	73.3	70.9	77
X-ray	54.6	55.3	53.5
CT	2.8	2.6	3.1
Ultrasonography	6.5	7.2	5.4
Associated injuries			
Any type	46.7	47.4	45.6
Cartilage	13	14.2	11.2
Medial meniscus	32.2	33.9	29.6
Lateral meniscus	27.7	29.9	24.1
LCL	10	9.9	10.1
MCL	13.7	13.2	14.6
PCL	4.3	4.1	4.6
Plan on returning to football			
Yes	60	58.3	62.6
No	40	41.7	37.4
Treatment planed			
Surgical	94.9	93.9	96.6
Non-surgical	5.1	6.1	3.4

## Discussion

The present study found that a high number of players reported a coach change before or during the season when they sustained their ACL injury, one in four for the males and one in three for the females. In male professional players, previous studies have found that the leadership style of the coach influences the injury burden of the team and that a high turnover of coaches leads to an increase of muscle injuries [7, 9]. The female players reported that 20.8% of the ACL injuries were sustained when the team changed coach during the season. Studies on the influence of a coach change on the risk of ACL injuries in amateur football are lacking but highly warranted given the results in the present study. Another notable founding that warrant a closer examination was the high number of ACL injuries in female players going to a higher division. An increased risk of ACL injury following a change to a higher division was previously only detected in a smaller group of elite male players [17].

For most of the variables examined in the present study, there were only small differences between male and female players in this large sample on football-specific epidemiological data on ACL injuries. Besides the expected gender differences in average height and weight, the females in the study were almost 5 years younger. This age difference is at least partly explained by female players retiring from football at a younger age than male players [22]. More male players reported that they were left-footed dominant and this is consistent with a meta-analysis on left-handedness and gender [18]. ACL injuries were more common in the early part of the game and nearly 1 in 4 occurred in the first 15 min. This is in line with previous studies and indicates that the ACL injury in football players is not primarily due to fatigue [6]. Preventive programs have proven to be effective in reducing the risk of non-contact ACL injuries in football [13, 15, 25, 26]. Given the increased risk during the early part of football games, implementing knee-specific exercises in the pre-game warm up could possibly help to further reduce the number of ACL injuries. The present study found that 31.4% of the females and only 15.6% of the male players reported that they use a knee-specific warm up program. This clearly indicate that even with the strong evidence of the effectiveness of the preventive programs, implementation and compliance remain a challenge. Continued efforts to promote neuro-muscular preventive programs are vital in reducing the number of ACL injuries in football. Although the frequencies differ between studies most have found that ACL injuries are more common in non-contact than contact situations [3, 5, 10, 24]. It is not easy to exactly define what a contact situation is in football, but in the present study, 6 out of 10 of the ACL injuries occurred in non-contact situations as defined by the players themselves. Previous studies have found that the incidents of ACL injuries are considerably higher during games compared to in training [5, 12, 24, 28]. In the present study, 66% of all ACL injuries happened during games. If the referee’s decision is used to assess the severity of the contact situation during games, only 0.5% resulted in a red card and 3.4% in a yellow card. Foul play should of course always be discouraged to protect the football players, but it does not appear like stricter rules would reduce the number of ACL injuries in football in a significant way. More ACL injuries occurred on artificial grass than on natural grass. Since the exposure to the different pitch surfaces was unknown, it could not be concluded if this difference was significant or not. However, given the advantages of artificial grass in densely populated areas or in cold climate regions, it would be unfeasible to convert artificial grass pitches to natural grass in an attempt to slightly reduce the risk of ACL injuries. Previous follow-ups on ACL reconstructed football players from the SNKLR found a return to football rate of 51–54%, and in this study, 60% of the players stated that they planned on returning to football [21, 23]. Consequently, the remaining 40% indicated that they did not intent to return to football. Although that number is in line with the previous studies on return to football in Sweden, the fact that they have made that decision before or shortly after the surgery when the questionnaire was filled out by many of the players is to our knowledge novel information and requires further investigation.

One obvious limitation in the present study and studies of this kind is of course the lack of exposure data or a matched control group. That limits the possibility for inference on the results obtained. Given the heterogenic and large group of football players and the number of variables included, the study is valuable in providing a foundation from which to develop hypothesis and ideas on how to reduce ACL injuries in football.

The present study highlights the need to further introduce and adhere to neuro-muscular training programs, and a special attention should be directed to teams changing the coach and players going to a higher division.

## Conclusions

Neuromuscular training programs have proven to reduce ACL injuries but greater adherence to these remain a challenge as only 1 in 5 of the ACL-injured football players report using them. Teams changing coach and players going to a higher division appear to have an increased risk of ACL injury warranting attention and further investigations.
